# Growth in milk consumption and reductions in child stunting: Historical evidence from cross-country panel data

**DOI:** 10.1016/j.foodpol.2023.102485

**Published:** 2023-07

**Authors:** Beliyou Haile, Derek Headey

**Affiliations:** a International Food Policy Research Institute, Environment and Production Technology Division, Eye Street, 1201 I St NW, Washington, DC 20005, United States; b International Food Policy Research Institute, Development Strategy and Governance Division, Eye Street, 1201 I St NW, Washington, DC 20005, United States

**Keywords:** Stunting, Milk, Dairy, Undernutrition, Food policy

## Abstract

Agricultural and food policies are increasingly being tasked with doing more to improve the nutritional status of low-income populations, especially reductions in child stunting. Which specific food sectors warrant additional policy attention is less clear, although a growing body of research argues that increased animal-sourced food consumption in general, and increased dairy consumption specifically, can significantly reduce the risks of stunting, as well as deficiencies in micronutrients and high quality protein. However, experimental research on dairy’s impacts on child growth in developing countries is very limited, and non-experimental evidence is confined to cross-sectional surveys. In this study we adopt a more macro lens by using a cross-country panel to show that increases in milk consumption over time are associated with large reductions in child stunting even after controlling for important confounding factors. Countries with high rates of stunting should therefore consider nutrition-sensitive strategies to increase dairy consumption among young children through both supply- and demand-side interventions.

## Highlights

• Dairy is rich in a range of nutrients and hypothesized to improve child growth.
• Dairy consumption is typically low in countries with high stunting prevalence.
• First study to analyze cross-country panel data on dairy and stunting reduction.
• Growth in dairy consumption is robustly associated with reduced stunting.
• Dairy development warrants greater attention in nutrition-sensitive food policies.

## 1 Introduction

Chronic undernutrition in early childhood – most often measured as linear growth failure, or stunting – is now widely recognized as having exceptionally high individual and social costs, including both child morbidity and mortality, delayed cognitive development and poor schooling outcomes, and lower productivity and wages in adulthood that ultimately retard economic growth (Behrman et al., 2004, Hoddinott et al., 2013). Growing cognizance of the vital importance of improving nutrition in early childhood has resulted in ambitious global targets, such as the World Health Assembly target of reducing under-5 stunting prevalence by 40% by 2025 (WHO, 2014), as well as the incorporation of stunting reduction in Sustainable Development Goal 2 (United Nations, 2015).

To achieve these targets, alternative investment options are being pursued that can broadly be classed as nutrition-specific or nutrition-sensitive. The former aims to tackle the immediate causes of fetal and child undernutrition through, for example, micronutrition supplementation for pregnant women and children, promotion of optimum breastfeeding and complementary feeding practices, and disease prevention and management*.* However, even nutrition experts estimate that scaling up the 10 most effective nutrition-specific interventions to 90% coverage in the highest undernutrition burden countries would only resolve 20% of that burden (Bhutta et al., 2013). This has led to calls for a much stronger role for nutrition-sensitive investments that address the critical underlying determinants of undernutrition, including food insecurity and poverty. A case in point is food and agriculture policies, which can impact multiple underlying determinants of undernutrition including household income, food security, and dietary quality (Ruel et al., 2018).

Within ministries of food and agriculture, however, the development of nutrition-sensitive agricultural strategies requires strategic decisions on which specific nutrient-dense foods to invest in for maximizing the sector’s contribution to nutritional improvement. For children, especially, there is a widespread consensus among nutritionists specializing in low and middle income country (LMIC) populations that animal-sourced foods (ASF) play a critical role in catalyzing healthier growth and cognitive development since ASFs are dense sources of both macronutrients – including high-quality protein – and a range of essential micronutrients that help promote both physical and cognitive development (Dror and Allen, 2011).

Among different ASFs, we argue that there is a particularly strong case to be made for the promotion of dairy products to help address child undernutrition. Biologically, dairy is rich in calories and high-quality proteins that are likely to address potentially critical amino acid deficiencies in infancy and early childhood (Dror and Allen, 2011). Dairy also provides several scarce and biologically important micronutrients such as calcium, vitamin A, riboflavin and vitamin B12, as well as a hormone called insulin-like growth factor-1 thought to play a critical role in programming linear growth, and perhaps cognitive development (Iannotti et al., 2013).

Empirically, evidence on the importance of dairy for reducing stunting – especially at a national scale – is surprisingly thin. Gold-standard randomized control trials (RCTs) that treat children with dairy products are often confined to high-income countries or older children less vulnerable to stunting (de Beer, 2012, Iannotti et al., 2013). A large multi-country analysis of Demographic Health Surveys (DHS) from 49 LMICs tested the association between stunting status and a child’s consumption of ASFs in the past 24 h (Headey et al., 2018). While that study found that children fed any ASF or multiple ASFs in the previous day had a lower risk of stunting, they also noted that dairy consumption had the strongest association with stunting reduction among all ASFs. Another recent literature looks at these associations with the aspiration of finding more causal evidence by exploiting the fact that cow-owning households in rural settings typically feed their infants dairy products on a higher frequency basis than households without cows due to the non-separability of production and consumption of dairy. These studies are quasi-experimental at best, using placebo tests (Hoddinott et al., 2015), matching techniques (Kabunga et al., 2017), potentially exogenous variation in lactation cycles (Choudhury and Headey, 2018) and exposure to livestock donation programs (Rawlins et al., 2014) to test whether better access to home-produced milk is associated with improve heights among pre-school children. Despite quite different methods, these studies reach similar conclusions, with household production of dairy products associated with a 0.5 standard deviation improvement in height-for-age z scores (HAZ).

However, these studies have important limitations. The datasets are cross-sectional and the regression analyses are still subject to concerns about endogeneity. The study samples are also confined solely to rural populations in East Africa and South Asia with at least some tradition of dairy production and consumption. This potentially limits the external validity of these findings since a large proportion of the developing world’s population is urban, and because many LMIC populations outside of East Africa and South Asia are lactose intolerant and have weak dairy traditions (Storhaug et al., 2017). Practically, policymakers in a wide and diverse range of countries need to know whether scaling up dairy consumption in their country – even if dairy is a non-traditional food – would lead to meaningfully large reductions in child stunting.

This is the question we aim to address in this study: are national-level increases in milk consumption associated with meaningful reductions in child stunting over time? To answer this question, we use a large and rich panel of countries, linking stunting estimates from repeated nationally representative surveys to measures of per capita dairy consumption from the Food and Agriculture Organization (FAO). We use country fixed effects regressions to focus on within-country variation in dairy consumption and stunting changes, exploiting the fact that dairy consumption has increased over time in many LMICs, often from a very low base. Most notable in this regard are East Asian countries – such as Japan, China, Vietnam and Thailand – that have witnessed rapid increases in dairy consumption despite very weak dairy traditions. Despite speculation that growth in dairy consumption contributed to the rapid declines in stunting prevalence in economically successful East Asian countries, such as Thailand (Heaver and Kachondam, 2002) and Japan (Takahashi, 1984), no empirical studies have investigated this possibility using rigorous statistical methods.

In this study we provide two important extensions to previous research on dairy consumption and stunting in developing countries. First, we extend the evidence from rural household surveys cited above (Choudhury and Headey, 2018, Hoddinott et al., 2015, Kabunga et al., 2017, Rawlins et al., 2014) to a much more global dataset for 91 LMICs, which allows us to draw conclusions with greater external validity. Second, the country-level fixed effects model we estimate allows us to control for country-level time-invariant stunting confounding factors. Implicitly, it also allows us to ask whether increases in dairy consumption at the national level are associated with nutritionally meaningful reductions in stunting, which is an important policy question for informing national strategies for nutrition, agriculture and trade. We are therefore able to test the contribution of growth in milk consumption for achieving reductions in child undernutrition, and to use these estimates to make cautious inferences on the potential for milk promotion strategies to meet ambitious global nutrition targets in the future.

## 2 Data and methods

### 2.1 Data sources and key variables

Our primary outcome indicator, child stunting prevalence, was drawn from the World Health Organization (WHO) Global Database on Child Growth and Malnutrition (De Onis and Blössner, 2003). This database compiles growth and malnutrition data from around the world since 1960 from population-based surveys based on probabilistic sampling. The other criteria for inclusion in the database are minimum sample size of 400 children and standard anthropometric measures need to be used. The WHO database provides estimates of prevalence of stunting among children less than 5 years of age based on height-for-age z-score (HAZ), which compares child’s height to the median child of the same sex and age in a healthy and well-nourished reference population. The WHO database uses the 2006 WHO study (WHO Multicentre Growth Reference Study Group and de Onis, 2006) as the reference population and defines a child as stunted if their HAZ is less than −2 standard deviations. Stunting prevalence of 40% or more indicates very high malnutrition severity while prevalence of 30–39%, 20–29%, and below 20% indicate, respectively, high, medium and low malnutrition severity (WHO, 1995).

We restricted the sample to 91 LMICs for which we have at least two data points for stunting prevalence. The panel is unbalanced with some countries having more observations than others, and the time gaps between observations also varies. See supplemental Table A1 for the list of study countries and years, along with stunting prevalence and nutrition data sources. The data cover five decades, of which 69% were collected after 2000, and 37% and 26% of observations pertain to sub-Saharan Africa and Latin America and the Caribbean (supplemental Table A2).

**Fig. 1:**
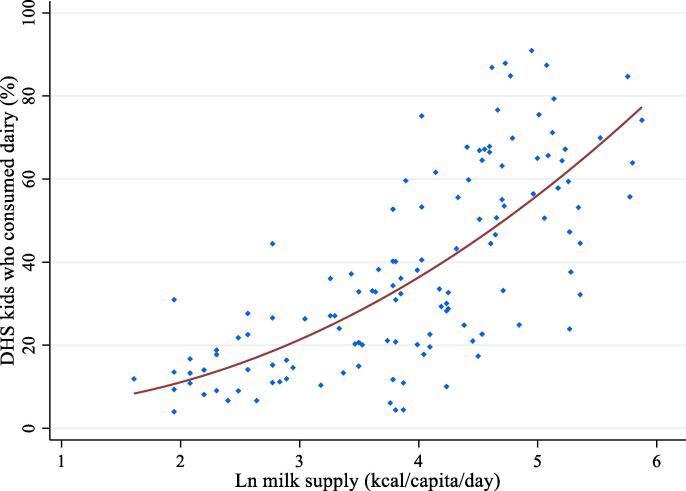
The Food Balance Sheet (FBS) indicator of milk supply per capita is strongly associated with the Demographic Health Survey measure of the prevalence of milk consumption among children 6–23 months in the past 24 h (%)*.* Note: Reported are scatterplots with plots of prediction values from a linear regression of the variable on the y-axis against the variable on the x-axis and its squared term. Ln refers to logarithmic transformation. FBS means Food Balance Sheet. Figure is based on a subsample of 67 countries for which DHS collected dietary data.

The WHO data on stunting prevalence were merged with FAO Food Balance Sheet (FBS) estimates of domestic food supply for human consumption, measured for different food groups, and reported in kilocalories per capita per day (k/c/d). The main variable of interest from the FBS was milk supply per capita, which captures the availability of energy from milk consumption (hereafter we follow the FAO terminology in referring to supply, even though this indicator is a proxy for consumption). The FBS has widely been used to analyze global and national food systems and changes in global food and nutrient supply (Beal et al., 2017, Bentham et al., 2020, Khoury et al., 2014, Wood et al., 2018), but is certainly measured with error. Out of concerns for such error, and because mean consumption per capita may differ from child-level milk consumption, we also examined the association between dairy consumption prevalence in the past 24 h among children 6–23 months of age from the Demographic and Health Surveys (DHS) and FBS milk supply to examine the association between FBS milk supply and child level consumption prevalence. As Fig. 1 shows, the association is indeed strong, with a correlation coefficient of 0.65 between FBS milk supply and percentage of DHS children consuming milk.

The multivariate analysis of the association between stunting prevalence and the log of milk supply controlled for several potentially confounding variables. These included four other nutrient-rich food groups from the FBS measured in k/c/d - non-dairy animal source foods (fish, meat, and eggs), fruits, vegetables, and legumes/nuts - as well as cereals grains and starchy roots and tubers. Average income – proxied by per capita GDP in constant 2010 US dollars – is an important control given the multiple ways in which economic growth could influence stunting and dietary changes (Headey, 2013, Smith and Haddad, 2002, Smith and Haddad, 2015). GDP per capita is sourced from the World Development Indicators (WDI) database (World-Bank, 2021). Additional WDI variables we control for include: 1) child dependency ratio, defined as the ratio between 0 and 14 years old pop and 15–69 years old pop expressed in percentages; 2) population growth rate, defined as $ln\left({\mathrm{pop}}_{t}/{\mathrm{pop}}_{t^{\text{'}}}\right)/\left(t-t^{\prime }\right)$*100, where t and $t^{\prime }$ (t > $t^{\prime }$) are consecutive years of available nutrition data; 3) the share of rural population with access to electricity; and 4) female labor force participation (15–24 years old). We also sourced estimates of population level access to improved sanitation facilities and improved drinking water from UNICEF (2023). Justifications for this selection of these control variables are provided below. Finally, for extensions and additional checks, we also use data on under-5 wasting prevalence from the same WHO nutrition database and the under-5 mortality rate (per 1,000 births) from the WDI as dependent variables. Pairwise correlations among the key variables of interest are shown in supplemental Table A3.

### 2.2 Statistical model

We modelled under-5 stunting prevalence in country *c* and period *t* (${\mathrm{Stunt}}_{\mathrm{ct}}$) as a function of logarithm (ln) milk supply in kcal/capita/day (k/c/d) ($ln{\mathrm{Milk}}_{\mathrm{ct}}$) whilst controlling for other sequentially added variables:

(1) $${\mathrm{Stunt}}_{\mathrm{ct}}=\alpha +\beta ln{\mathrm{Milk}}_{\mathrm{ct}}+\;\Phi^{\text{'}}{Food}_{\mathrm{ct}}+\Pi^{\text{'}}{Economic}_{\mathrm{ct}}+\Sigma^{\text{'}}{WASH}_{\mathrm{ct}}+T{+\varepsilon }_{\mathrm{ct}}$$

Where Food is a matrix of the supply (in k/c/d) of the four control food groups (non-dairy ASFs, fruits, vegetables, legumes/nuts, cereal grains, and starchy roots and tubers). Economic is a matrix of economic indicators that may affect stunting including ln GDP per capita measured in constant 2010 USD, population growth rate, ln child dependency ratio, ln population with access to electricity expressed in percentages, and ln of female labor force participation rate (15–24 years old). WASH consists of ln of population using improved sanitation facilities and ln of population using improved drinking water sources, both expressed in percentages. T consists of fixed effects for decades and subregion-specific decadal trends. The decade variable takes four values with nutrition data captured in the 1970s & 1980s combined into one group (due to small sample size) and other values capturing nutrition data collected in the 1990s, 2000s, and 2010s. The eight subregions covered in our study include five in Asia (central, eastern, south-eastern, southern, and western), two in Africa (North Africa and sub-Saharan Africa) and Latin America and the Caribbean.

These control variables were selected to minimize the influence of important confounding factors. The effect of dairy consumption on stunting would be inflated if we failed to control for consumption of other nutrient-dense foods that potentially reduce stunting (Headey et al., 2018). Dairy is also an income-elastic good whose consumption rises with income (Colen et al., 2018), so controlling for GDP per capita is important, along with electricity to capture a dimension of household wealth as well as a nutritionally relevant infrastructure development (Fujii et al., 2018).

Demographic variables have been shown to influence stunting, with higher fertility and child dependency rates linked to elevated risks of stunting in survey-based regression analysis (Headey et al., 2015). There is also some observational and experimental evidence that shows significant associations between stunting and improved WASH conditions (Cumming and Cairncross, 2016, Freeman et al., 2017, Hammer and Spears, 2016, Hathi et al., 2017, Headey and Palloni, 2019), although the experimental evidence is weaker than the observational evidence. We control for female labor force participation as another potential factor that could influence mother’s decisions on whether to breastfeed or provide cow’s milk or infant formula, but which could also influence stunting through other mechanisms related to care practices and women’s income and empowerment.

The model error term is captured by ε, and we cluster standard errors from all regressions at country level (White, 1980). Given the cross-country variation in the size of under-5 population, we estimate Equation (1) with^1^ and without weighting the regressions by under-5 population and report the latter in the supplemental material. In addition to the regression analysis described above, we also explore bivariate associations between core variables of interest using scatter plots and predicted values from linear models.

As robustness checks, we first estimate a version of Equation (1) using two alternative dependent variables: under-5 wasting prevalence and under-5 mortality rates. While there is micro evidence linking child milk consumption with lower risk of stunting, to our knowledge milk consumption has not been shown to reduce child wasting in LMICs and so we do not expect a statistically significant association between the two. Similarly, while under-5 children with severe acute malnutrition (e.g. wasting) face a significantly higher risk of mortality (Olofin et al., 2013), the association between stunting and mortality is weaker, and we do not know of any expect a significant association between milk consumption and under-5 mortality. Moreover, the fact that preterm birth complications, birth asphyxia, pneumonia, congenital anomalies, diarrhea, and malaria are the leading causes of under-5 mortality (UN-IGME, 2021), suggests that milk consumption should not have a significant association with mortality reduction, unless the association is spurious. Hence, we consider this mortality regression as akin to a placebo test.

## 3 Descriptive statistics

Table 1 shows the regional sample distribution and means of the key variables used in this study. South-East Asia, Southern Asia and SSA have the highest stunting prevalence while stunting is the lowest for Eastern Asia. South-eastern Asia and SSA also have the lowest per capita milk supply, suggesting low milk consumption could play some role in explaining high stunting rates in these regions. South Asia is potentially something of an outlier insofar as most countries in the region – including India – have relatively high levels of milk consumption but also very high rates of stunting. However, poor child health, extremely poor WASH conditions, and other dietary factors could explain India’s unusually high stunting rate despite relatively widespread dairy consumption (Headey et al., 2016, Headey and Palloni, 2020, Nguyen et al., 2018, Spears, 2018). Moreover, dairy consumption children in countries such as India remains far from universal: the 2015–16 DHS suggested only half of children consumed dairy in the past 24 h.

**Table 1:** Stunting prevalence, per capita milk supply and per capita GDP by region for 91 LMICs.

Region	No. of countries	No. of observations	Average stunting prevalence (%)	Average milk supply (k/c/d)	Average per capita GDP (constant 2010 US $)
	(1)	(2)	(3)	(4)	(5)
Central Asia	4	12	16	256	4917
Eastern Asia	2	18	9	152	4619
LAC	19	138	16	146	5630
Northern Africa	5	30	19	158	3238
South-eastern Asia	9	61	31	30	3296
Southern Asia	7	35	28	137	3137
Sub-Saharan Africa	37	193	31	60	1716
Western Asia	8	37	18	189	5146
Total	91	524			
Note: LAC = Latin America and the Caribbean consisting of Central America, South America and Central America. Eastern Asia includes China and Mongolia. k/c/d stands for kilocalories per capita per day. Statistics in columns 4, 5, and 6 are based on the most recent year for which stunting data are available for study countries.					

**Fig. 2:**
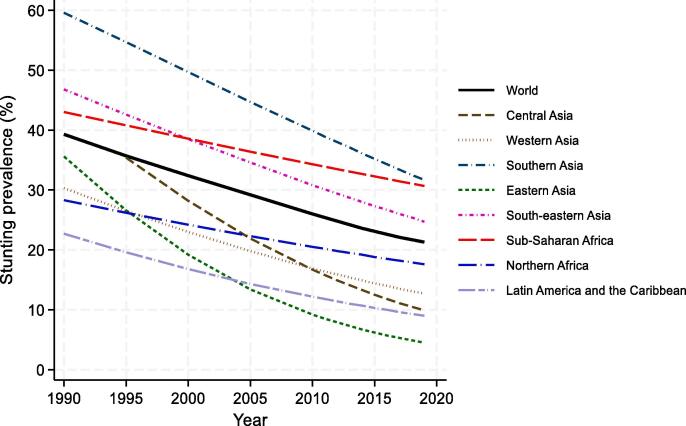
Regional and temporal trends in stunting prevalence Source: Authors’ calculation based on regional stunting data from UNICEF, WHO, World Bank Group Joint Malnutrition Estimates, March 2020 Edition. Eastern Asia and South-eastern Asia excludes Japan.

In terms of trends, most regions have seen reductions in stunting prevalence over time, and indeed SSA was the only region to experience relatively limited reduction in stunting prevalence (Fig. 2). This trend, coupled with SSA’s relatively high fertility rate with a weaker decline over the years, has resulted in an increase in the absolute number of stunted children from 40.2 million in 1990 to 52.4 million in 2019 (30% increase) given region’s fertility rate with a relatively weak decline through the years, has resulted in an.

**Fig. 3:**
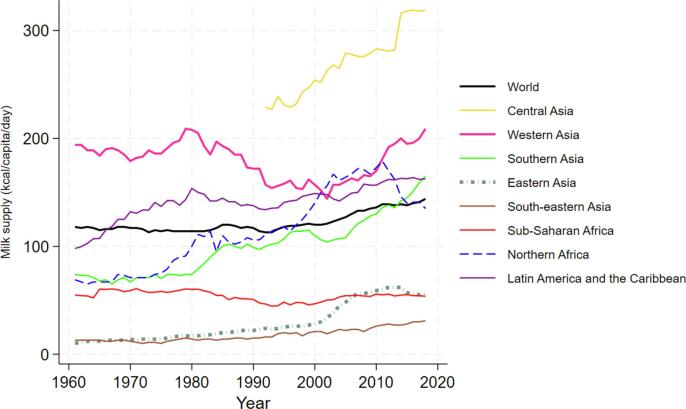
Regional and temporal trends in milk supply Source: Authors’ own calculation based on regional data from UN FAO Food Balance Sheet. Milk supply data for pre 1990 years not available for Central Asia.

All study regions except SSA also experienced increases in milk supply since 1960 (Fig. 3). In the past 30 years some of the most rapid growth in milk consumption has occurred in Southeast Asia (Vietnam, Thailand) and China, as well as Central Asia since the break-up of the USSR, and South Asia, particularly India. See supplemental Table A4 for ranking of countries based on average annual growth rate of milk supply between the first and last years of available nutrition data.

**Fig. 4:**
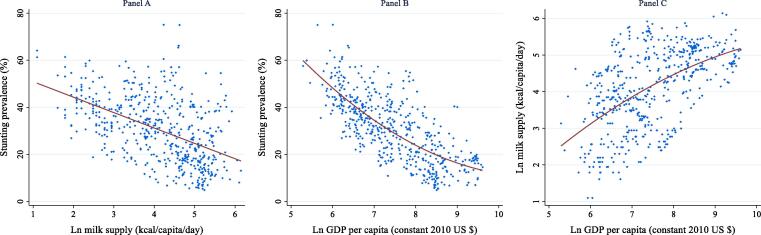
Cross-sectional associations between stunting prevalence, per capita milk supply and per capita GDP Note: Reported are scatterplots with plots of prediction values from a linear regression of the variable on the y-axis against the variable on the x-axis and its squared term. Ln refers to logarithmic transformation.

Fig. 4 examines bivariate associations between stunting prevalence, per capita milk supply and per capita GDP. Examining the associations between stunting and milk consumption with GDP per capita is important because income is likely the most important confounding factor in any non-experimental study assessing associations between stunting and milk consumption, as micro-econometric studies show that milk is an income-elastic good whose consumption tends to increase markedly with rising household incomes (Colen et al., 2018). Hence, since economic growth is a well-known predictor of stunting reduction (Headey, 2013, Smith and Haddad, 2002, Smith and Haddad, 2015), it is critically important to control for economic growth in any cross-country regression analysis of stunting and milk consumption.

As expected, milk supply and GDP are strongly and negatively associated with stunting (Fig. 4, Panels A and B), while the association between GDP and milk supply is also positive, as expected (Fig. 4, Panel C). Also apparent from the scatterplots is the presence of some degree of heteroskedasticity; in particular, countries with higher levels of milk supply still display large variations in stunting rates. This is not surprising as there are many non-dairy and non-dietary determinants of stunting, but it emphasizes the importance of multivariate tests that control for various confounding factors. It is likewise also important to control for country fixed effects, especially as dairy production and consumption traditions are strongly associated with climate, location and other time-invariant factors that could influence stunting.

## 4 Regression results

Table 2 reports fixed effects regressions of stunting prevalence as a function of the log of per capita milk supply, with different sets of controls, also specified in logarithmic terms. Since all right-hand side variables are measured in logs and the models are fixed effects, the coefficients in effect represent the predicted change in stunting prevalence resulting from proportional changes in the various explanatory variables.

**Table 2:** Population-weighted fixed effects estimates of the association between milk supply and stunting prevalence, controlling for different sets of potentially confounding factors.

	(1)	(2)	(3)	(4)
Ln milk supply (k/c/d)	−6.82***	−6.60***	−3.18***	−3.18***
	(1.20)	(1.10)	(1.14)	(1.18)
Ln non-dairy animal-source foods (k/c/d)		−0.35	−0.25	−0.20
		(1.06)	(0.83)	(0.85)
Ln fruit supply (k/c/d)		−0.40	−0.68	−0.83
		(0.74)	(0.62)	(0.62)
Ln vegetable supply (k/c/d)		−2.52*	−1.30	−1.44
		(1.50)	(1.49)	(1.52)
Ln legume and nuts supply (k/c/d)		0.52	0.49	0.58
		(0.59)	(0.61)	(0.62)
Ln starchy roots supply (k/c/d)		−0.96	−0.28	−0.16
		(1.26)	(1.11)	(1.12)
Ln cereals supply (k/c/d)		0.90**	0.58**	0.59**
		(0.35)	(0.29)	(0.27)
Ln GDP per capita (constant 2010 US $)			−6.70***	−6.15***
			(1.61)	(1.56)
Population growth rate			0.01	0.03
			(0.22)	(0.21)
Ln child dependency ratio			11.39***	11.18***
			(3.32)	(3.11)
Ln access to electricity (% of population) (rural)			−1.33*	−1.06
			(0.77)	(0.81)
Ln of female labor force participation rate (15–24) (%)				1.12
				(2.28)
Ln of population using improved sanitation (%)				−0.33
				(0.33)
Ln of population using improved drinking water (%)				−3.86
				(2.66)
Number of observations	524	524	524	524
Adjusted R2	0.893	0.899	0.911	0.911
Adjusted R2 within	0.13	0.17	0.27	0.27
Note: Reported are fixed effects estimates where regressions are weighted by under-five population. Ln means natural logarithm. k/c/d stands for kilocalories per capita per day. Robust standard errors clustered at the country level in parentheses. All columns control for subregion-specific time trends. *** p < 0.01, ** p < 0.05, * p < 0.1.				

Regression (1) reports the most basic country fixed effects model (corresponding, approximately, to Panel A of Fig. 4), confirming a strong and highly significant negative association between changes in per capita milk supply and changes in stunting prevalence: a 10% increase in milk supply is associated with a 0.68 point reduction in stunting prevalence. Regression (2) controls for changes in the per capita supply of staples and other non-staple nutrient-rich food groups recorded in the FAO Food Balance Sheets. The coefficient on milk supply is only slightly reduced. Regression (3) adds GDP per capita, population growth rate, child dependency ratio and access to electricity to the model. Given the strong inter-relationships between stunting, milk supply and GDP (Fig. 4), it is not surprising to see a marked reduction in the coefficient of the log of milk supply, with a 10% increase in milk supply associated with a 0.31 point reduction in stunting. Unsurprisingly given previous studies on economic growth and stunting (Headey, 2013, Smith and Haddad, 2002, Smith and Haddad, 2015), the coefficient on the log of GDP per capita is negative, highly statistically significant, and large in magnitude. The child dependency ratio is also strongly associated with stunting, suggesting worse nutritional outcomes for children in societies where families are larger and parental and societal resources for children are spread thin. Controlling for additional covariates that capture access to quality drinking water, sanitation facilities, and female labor force participation does not affect the coefficient of milk (column 4).

Also encouraging are the model fitness statistics reported at the bottom of Table 2, which show that adding controls to the model progressively improves the explanatory power of the model from 13% to 27% in the case of within-country variation. These increases suggest that our controls are adding substantial explanatory power to the model. Fixed effects results from un-weighted regressions of stunting and milk consumption are similar to results reported in Table 2 (see supplemental Table A5). Finally, as expected, we do not find statistically significant associations between milk consumption on the one hand and under-5 wasting and mortality rates on the other (supplemental Table A6).

## 5 Discussion

Although stunting rates have declined in most regions of the world in recent decades, they remain unacceptably high in much of Asia and Africa (with the population of stunted children rising in Africa), and the COVID-19 crisis has almost certainly seriously reversed these recent gains (Headey et al., 2020). Catalyzing faster progress against stunting will require scaling up proven nutrition-specific interventions (Bhutta et al., 2013, Osendarp et al., 2021) as well as investments and policies that promote nutrition-sensitive agriculture and food systems.

A number of prominent nutritionists have long argued that dairy is highly effective in reducing stunting (de Beer, 2012, Drewnowski, 2017, Dror and Allen, 2011, Dror and Allen, 2014, Michaelsen, 2013, Visioli and Strata, 2014), and dairy has long been a part of school feeding programs in some LMICs (Iannotti et al., 2013). While there has been a very strong push for early childhood dairy consumption in the developed worked, there is still an underappreciation of the critical role that dairy development could play in redressing stunting in *early* childhood across the developing world. Previous research in agricultural economics has demonstrated the importance of household milk production for reducing stunting among rural children in cross-sectional studies, but to our knowledge this is the first study to demonstrate that recent historical increases in milk consumption at the national level is associated with meaningful reductions in stunting in a large and diverse panel of LMICs.

There are limitations to this kind of cross-country analysis. Measurement error is a concern with FAOSTAT data, although we did show that the log of per capita milk consumption is strongly associated with child level consumption of dairy products in the past 24 h from the DHS. Endogeneity is also a concern, although the robustness of the dairy coefficient to the inclusion of other foods and GDP per capita as controls constitutes relatively compelling evidence, especially given the broad consistency of our results with evidence from limited experimental and observational studies, as reviewed by Choudhary and Headey (2018).

Collectively, we argue that this body of evidence warrants a much greater prioritization of dairy development in national food and nutrition strategies. Dairy development strategies need to be adapted to local circumstances, considering factors like the agro-ecological potential for dairy production (which is influence by temperature and livestock diseases) as well as whether a population has strong traditions or strong demand for dairy. In countries with weaker traditions, there are important success stories in South-East Asia which other countries can learn from. While rising incomes and Westernization of diets are incontrovertible demand-side drivers of rising dairy consumption in Asian countries like Vietnam and Thailand, there are also indications of public policies playing a key role in increasing consumption of dairy products (Heaver and Kachondam, 2002, Nguyen et al., 2021). In general, Asian countries have relied on sustained and sequenced support to the nascent dairy industry combined with flexible trade policies designed to bolster demand not met by domestic supply. These kinds of hybrid supply-side strategies, where imports continue to play an important role even after sustained growth in the domestic dairy sector, are particularly important for tropical developing countries with climates that inherently limited domestic production potential. However, countries such as Thailand also stabilized and catalyzed more demand for dairy products through demand-side interventions as well, notably a school feeding program that consumed 30–40% of domestically produced milk (Heaver and Kachondam, 2002).

In LMICs with stronger traditions of both dairy production and consumption (notably South Asia and Eastern Africa), taking steps to modernize dairy production and marketing is critical. In these countries dairy herds are large in aggregate, but very small at the household level, often highly subsistence oriented, and highly unspecialized in the sense that cattle are used to provide milk but also traction, transport and other services. Expanding production and consumption is certainly possible in these circumstances, as India has shown (Cunningham, 2009), but requires a combination of expanding access to markets (e.g. collection centers, dairy cooperatives, contract farming), improving the genetic makeup of the livestock herd, providing access to veterinary services, scaling up processing and storage technologies, building a suitable business environment for commercial dairy firms, and providing appropriate regulation and monitoring of food safety both for public health reasons as well as consumer trust. A number of recent studies from East Africa highlight the complexities of these challenges in agrarian settings where the productivity and commercialization of the dairy sector is low, but gradually improving (Minten et al., 2023, Minten et al., 2020, Van Campenhout et al., 2021, Vandercasteelen et al., 2021), while one recent study also shows that nutrition-oriented behavioral change communications can bolster the impact of smallholder dairy development programs on children’s consumption of dairy products (Flax et al., 2021).

Given that milk consumption is remarkably low in so much of sub-Saharan Africa and South-East Asia – and still low in large pockets of South Asia – there is clearly tremendous scope for public policies and public–private partnerships to scale up dairy consumption, especially among pre-school populations. However, it also behooves us here to address several important objections to this recommendation.

First, there are some concerns that promotion of dairy among infants and young children could displace breastfeeding or lead to increased promotion of milk-based breastmilk substitutes (Baker et al., 2021). Although cow’s milk is often used in infant formulas, which often displace breastfeeding, we argue that this fact should not be used to discourage consumption of cow’s milk by young children. It is certainly true that cow’s milk is not a substitute for breastmilk, but dairy products can be a very beneficial complementary food after the approximately 6 months of age when breastmilk alone can no longer provide complete nutrition for infants. To our knowledge, evidence on cow’s milk being used as a substitute for breastmilk in developing countries is very limited (Choudhury and Headey, 2018), and more research is needed on whether increased availability of cow’s milk leads to breastmilk substitution. One potential implication is that dairy promotion needs to be strongly accompanied by appropriate nutrition education campaigns to promote exclusive breastfeeding, which have indeed been trialed in countries such as Vietnam (Nguyen et al., 2017).

Second, it is often claimed that dairy production involves high greenhouse gas (GHG) emissions. However, an authoritative study (Poore and Nemecek, 2018) that provided estimates of GHGs per unit of calories produced showed that milk produced fewer GHGs that rice, farmed fish, olive oil, and produces just 5% of the GHGs per kilogram of food that beef produces (milk’s low unit costs are even more accentuated when measure in GHGs per unit of protein). These differences stem from the fact that specialized dairy cows produce milk on an almost continuously productive basis over a long period of time, making their conversion of grazing land and animal feed into food (milk) far more efficient than beef.

Third, lactose intolerance is often raised as a potential obstacle to increasing dairy consumption in tropical countries. However, lactose intolerance emerges in later childhood and adulthood, and is generally not an issue for infants and young children, who produce adequate amount of lactase, the enzyme needed to adequate digest milk (Heyman, 2006). Moreover, older children and adults, even in lactose-intolerant populations, can generally consume modest amounts of dairy without digestive issues. Indeed, the rapid increases in dairy consumption in countries such as Japan, China, Thailand and Vietnam show that lactose intolerance is not an important barrier to increasing milk consumption among children, who can clearly benefit from the high nutrient density of dairy products.

We believe this study, along with the existing body of evidence linking milk consumption to reduced risks of stunting, justifies greater investments in dairy production – and potentially trade reforms – for the objectives of reducing stunting risks as well as micronutrient deprivation in developing countries. Ambitiously scaling up dairy consumption in developing countries will require a combination of stronger international support for dairy development projects, sequenced and sustained domestic policy support balanced with pragmatic trade policies (especially in countries with lower dairy production potential), public–private partnerships to leverage common incentives to expand milk consumption, and closer multi-sectoral collaborations within governments to better integrate food and nutrition policies for the dairy sector. With these ingredients, the scope for dairy development to make a meaningful contribution to the eradication of stunting is prodigious.

**Data availability**

All data used in this study are publicly available and are listed below. Researchers interested in replicating the study should contact the authors.

• National-level malnutrition data: World Health Organization (https://www.who.int/nutgrowthdb/database/en/)
• Regional-level malnutrition data: UNICEF, WHO, World Bank Group Joint Malnutrition Estimates, March 2020 Edition. https://data.unicef.org/resources/dataset/malnutrition-data/
• Access to improved drinking water and sanitation: UNICEF 2023. UNICEF Data Warehouse. https://data.unicef.org/resources/data_explorer/unicef_f/
• National and regional-level supply of milk and other food groups: Food and Agriculture Organization of the United Nations (FAOSTAT): https://www.fao.org/faostat/en/#data/FBS
• Dairy consumption among children: Demographic and Health Surveys (DHS) https://dhsprogram.com/
• GDP in constant 2010 US dollars: The World Bank. https://data.worldbank.org/indicator/NY.GNP.PCAP.KD
• Population (historical and projected) and child dependency ratio: United Nations, Department of Economic and Social Affairs, Population Division (2019). World Population Prospects 2019, Online Edition. Rev. 1. https://population.un.org/wpp/Download/Standard/Population/
• Country classifications based on income level (GNI per capita) from the World Bank
• https://datahelpdesk.worldbank.org/knowledgebase/articles/906519-world-bank-country-and-lending-groups
• Under five morality rates, share of rural population with access to electricity, and female labor force participation: The World Development Indicators (WDI). https://data.worldbank.org/indicator/SP.DYN.IMRT.IN
• Greenhouse gas emission across the supply chain. https://ourworldindata.org/

## Declaration of Competing Interest

The authors declare that they have no known competing financial interests or personal relationships that could have appeared to influence the work reported in this paper.

## Footnotes

1 Implemented using *reghdfe* command in Stata that estimates a linear regression model absorbing fixed effects for countries as well as region-specific time trends.
