# Supplementary material for: Growth in milk consumption and reductions in child stunting: Historical evidence from cross-country panel data
Source: Food Policy. 2023 Jul;118:102485. doi: 10.1016/j.foodpol.2023.102485 (PMC10400883; doi:10.1016/j.foodpol.2023.102485)
Supplement: Supplementary data 1 [file mmc1.docx]

**Supplemental material: Growth in milk consumption and reductions in child stunting: Historical evidence from cross-country panel data**

Contents

[Table A1. Stunting prevalence and data sources 2](#_Toc134901748)

[Table A1. Stunting prevalence and data sources (Cont’d) 3](#_Toc134901749)

[Table A1. Stunting prevalence and data sources (Cont’d) 4](#_Toc134901750)

[Table A1. Stunting prevalence and data sources (Cont’d) 5](#_Toc134901751)

[Table A1. Stunting prevalence and data sources (Cont’d) 6](#_Toc134901752)

[Table A1. Stunting prevalence and data sources (Cont’d) 7](#_Toc134901753)

[Table A2. Sample composition by region and decades covered 8](#_Toc134901754)

[Table A3. Correlation coefficients between key variables 9](#_Toc134901755)

[Table A4. Country ranking based on compound average growth rate (CAGR) of milk supply 11](#_Toc134901756)

[Table A4. Country ranking based on compound average growth rate (CAGR) of milk supply (Cont’d) 12](#_Toc134901757)

[Table A5. Fixed effects estimates of the association between milk supply and stunting prevalence (unweighted regressions) 13](#_Toc134901758)

[Table A6. Fixed effects estimates of the association between milk supply, wasting prevalence, and under-5 mortality 14](#_Toc134901759)

## Table A1. Stunting prevalence and data sources

|  |  |  |  |  |  |  |  |  |
| --- | --- | --- | --- | --- | --- | --- | --- | --- |
| Country | Year | Stunting  prevalence (%) | Data  source |  | Country | Year | Stunting  prevalence (%) | Data  source |
| Algeria | 1992 | 23 | PAPFAM |  | Madagascar | 1992 | 61 | DHS |
| Algeria | 1995 | 23 | ONS |  | Madagascar | 1995 | 55 | MICS |
| Algeria | 2000 | 24 | MICS |  | Madagascar | 2004 | 53 | DHS |
| Algeria | 2002 | 24 | ONS |  | Malawi | 1992 | 55 | DHS |
| Algeria | 2005 | 16 | MICS |  | Malawi | 2000 | 55 | DHS |
| Algeria | 2013 | 12 | MICS |  | Malawi | 2005 | 52 | DHS |
| Angola | 2001 | 51 | MICS |  | Malawi | 2006 | 53 | MICS |
| Angola | 2016 | 38 | DHS |  | Malawi | 2010 | 47 | DHS |
| Armenia | 1998 | 15 | NNS |  | Malawi | 2014 | 42 | ONS |
| Armenia | 2001 | 17 | DHS |  | Malawi | 2016 | 37 | DHS |
| Armenia | 2005 | 18 | DHS |  | Malaysia | 1999 | 21 | NNS |
| Armenia | 2010 | 21 | DHS |  | Malaysia | 2015 | 18 | ONS |
| Armenia | 2016 | 9 | DHS |  | Malaysia | 2016 | 21 | ONS |
| Azerbaijan | 2000 | 24 | MICS |  | Mali | 2001 | 42 | DHS |
| Azerbaijan | 2006 | 27 | DHS |  | Mali | 2006 | 38 | DHS |
| Azerbaijan | 2011 | 16 | ONS |  | Mali | 2010 | 28 | MICS |
| Azerbaijan | 2013 | 18 | NNS |  | Mali | 2013 | 38 | DHS |
| Bangladesh | 1997 | 60 | DHS |  | Mali | 2015 | 30 | MICS |
| Bangladesh | 2000 | 51 | DHS |  | Mauritania | 1988 | 40 | LSMS |
| Bangladesh | 2004 | 50 | DHS |  | Mauritania | 1991 | 55 | PAPFAM |
| Bangladesh | 2007 | 43 | DHS |  | Mauritania | 1996 | 50 | MICS |
| Bangladesh | 2011 | 41 | DHS |  | Mauritania | 2001 | 39 | DHS |
| Bangladesh | 2013 | 42 | MICS |  | Mauritania | 2007 | 31 | MICS |
| Bangladesh | 2014 | 36 | DHS |  | Mauritania | 2008 | 20 | NNS |
| Belize | 2006 | 22 | MICS |  | Mauritania | 2015 | 28 | MICS |
| Belize | 2011 | 19 | MICS |  | Mauritius | 1985 | 27 | NNS |
| Belize | 2016 | 15 | MICS |  | Mauritius | 1995 | 14 | NNS |
| Benin | 2001 | 36 | DHS |  | Mexico | 1988 | 27 | NNS |
| Benin | 2006 | 43 | DHS |  | Mexico | 1989 | 40 | NNS |
| Benin | 2014 | 34 | MICS |  | Mexico | 1996 | 40 | NNS |
| Bolivia | 1981 | 49 | NNS |  | Mexico | 1999 | 21 | NNS |
| Bolivia | 1998 | 33 | DHS |  | Mexico | 2006 | 15 | NNS |
| Bolivia | 2004 | 32 | DHS |  | Mexico | 2012 | 14 | NNS |
| Bolivia | 2008 | 27 | DHS |  | Mexico | 2015 | 12 | MICS |
| Bolivia | 2012 | 18 | ONS |  | Mongolia | 1997 | 31 | NNS |
| Bolivia | 2016 | 16 | DHS |  | Mongolia | 1999 | 30 | NNS |
| Botswana | 1996 | 35 | ONS |  | Mongolia | 2000 | 30 | MICS |
| Botswana | 2000 | 29 | MICS |  | Mongolia | 2005 | 27 | MICS |
| Botswana | 2008 | 29 | ONS |  | Mongolia | 2010 | 15 | NNS |
| Brazil | 1996 | 13 | DHS |  | Mongolia | 2013 | 11 | MICS |
| Brazil | 2007 | 7 | ONS |  | Morocco | 1987 | 31 | DHS |
| Brazil | 2014 | 13 | SVLLS |  | Morocco | 1992 | 30 | DHS |
| Burkina Faso | 1993 | 39 | DHS |  | Morocco | 1997 | 29 | PAPFAM |
| Burkina Faso | 1999 | 41 | DHS |  | Morocco | 2004 | 23 | DHS |
| Burkina Faso | 2003 | 43 | DHS |  | Morocco | 2011 | 15 | PAPFAM |
| Burkina Faso | 2006 | 40 | MICS |  | Mozambique | 1995 | 60 | MICS |
| Burkina Faso | 2009 | 35 | NNS |  | Mozambique | 2001 | 50 | ONS |
| Burkina Faso | 2010 | 35 | DHS_MICS |  | Mozambique | 2003 | 47 | DHS |
| Burkina Faso | 2011 | 35 | NNS |  | Mozambique | 2008 | 44 | MICS |
|  |  |  |  |  | Mozambique | 2011 | 43 | DHS |
|  |  |  |  |  |  |  |  |  |

Source: World Health Organization, Global Dataset of Child Stunting

## Table A1. Stunting prevalence and data sources (Cont’d)

|  |  |  |  |  |  |  |  |  |
| --- | --- | --- | --- | --- | --- | --- | --- | --- |
| Country | Year | Stunting prevalence (%) | Stunting data source |  | Country | Year | Stunting prevalence (%) | Stunting data source |
| Burkina Faso | 2012 | 33 | NNS |  | Myanmar | 2000 | 41 | MICS |
| Burkina Faso | 2013 | 31 | NNS |  | Myanmar | 2003 | 41 | MICS |
| Burkina Faso | 2014 | 29 | NNS |  | Myanmar | 2010 | 35 | MICS |
| Burkina Faso | 2016 | 27 | NNS |  | Myanmar | 2016 | 29 | DHS |
| Cambodia | 1996 | 59 | ONS |  | Namibia | 1992 | 36 | DHS |
| Cambodia | 2000 | 49 | DHS |  | Namibia | 2000 | 29 | DHS |
| Cambodia | 2006 | 43 | DHS |  | Namibia | 2007 | 29 | DHS |
| Cambodia | 2008 | 40 | NNS |  | Namibia | 2013 | 23 | DHS |
| Cambodia | 2011 | 40 | DHS |  | Nepal | 1975 | 75 | NNS |
| Cambodia | 2014 | 32 | DHS |  | Nepal | 2001 | 57 | DHS |
| Cameroon | 1991 | 32 | DHS |  | Nepal | 2006 | 49 | DHS |
| Cameroon | 2004 | 36 | DHS |  | Nepal | 2011 | 41 | DHS |
| Cameroon | 2006 | 37 | MICS |  | Nepal | 2014 | 37 | MICS |
| Cameroon | 2011 | 33 | DHS/MICS |  | Nepal | 2017 | 36 | DHS |
| Cameroon | 2014 | 32 | MICS |  | Nicaragua | 1993 | 30 | LSMS |
| Cape Verde | 1983 | 23 | NNS |  | Nicaragua | 1998 | 30 | LSMS |
| Cape Verde | 1994 | 21 | ONS |  | Nicaragua | 2001 | 25 | DHS |
| Central African Rep. | 2000 | 44 | MICS |  | Nicaragua | 2007 | 23 | ONS |
| Central African Rep. | 2006 | 43 | MICS |  | Nicaragua | 2012 | 17 | ONS |
| Central African Rep. | 2011 | 41 | MICS |  | Niger | 1985 | 44 | ONS |
| Chad | 1997 | 45 | DHS |  | Niger | 1992 | 45 | DHS |
| Chad | 2004 | 44 | DHS |  | Niger | 2000 | 53 | MICS |
| Chad | 2010 | 39 | MICS |  | Niger | 2006 | 55 | DHS/MICS |
| Chad | 2015 | 40 | DHS/MICS |  | Niger | 2012 | 44 | DHS/MICS |
| China | 1987 | 38 | NNS |  | Nigeria | 2007 | 39 | MICS |
| China | 1990 | 32 | SVLLS |  | Nigeria | 2008 | 41 | DHS |
| China | 1992 | 38 | NNS |  | Nigeria | 2011 | 36 | MICS |
| China | 1995 | 31 | SVLLS |  | Nigeria | 2013 | 36 | DHS |
| China | 1998 | 20 | SVLLS |  | Nigeria | 2014 | 22 | NNS |
| China | 2000 | 18 | SVLLS |  | Nigeria | 2015 | 33 | NNS |
| China | 2002 | 22 | NNS |  | Nigeria | 2017 | 44 | MICS |
| China | 2005 | 12 | SVLLS |  | Pakistan | 1991 | 55 | DHS |
| China | 2008 | 14 | SVLLS |  | Pakistan | 1994 | 43 | ONS |
| China | 2009 | 13 | SVLLS |  | Pakistan | 2001 | 42 | NNS |
| China | 2010 | 9 | SVLLS |  | Pakistan | 2011 | 43 | NNS |
| China | 2013 | 8 | NNS |  | Pakistan | 2013 | 45 | DHS |
| Colombia | 1980 | 28 | NNS |  | Panama | 1980 | 24 | NNS |
| Colombia | 1989 | 22 | NNS |  | Panama | 1997 | 17 | LSMS |
| Colombia | 1995 | 20 | DHS |  | Panama | 2003 | 24 | NNS |
| Colombia | 2000 | 18 | DHS |  | Panama | 2008 | 19 | NNS |
| Colombia | 2005 | 16 | DHS |  |  |  |  |  |
| Colombia | 2010 | 13 | DHS |  |  |  |  |  |
| Congo (Brazzaville) | 1987 | 34 | NNS |  |  |  |  |  |
| Congo (Brazzaville) | 2005 | 31 | DHS |  |  |  |  |  |
| Congo (Brazzaville) | 2012 | 24 | DHS |  |  |  |  |  |
| Congo (Brazzaville) | 2015 | 21 | MICS |  |  |  |  |  |
|  |  |  |  |  |  |  |  |  |

## Table A1. Stunting prevalence and data sources (Cont’d)

|  |  |  |  |  |  |  |  |  |
| --- | --- | --- | --- | --- | --- | --- | --- | --- |
| Country | Year | Stunting prevalence  (%) | Stunting  data  source |  | Country | Year | Stunting  prevalence  (%) | Stunting  data  source |
| Côte d'Ivoire | 1986 | 23 | NNS |  | Paraguay | 1990 | 18 | DHS |
| Côte d'Ivoire | 1999 | 31 | DHS |  | Paraguay | 2005 | 18 | NNS |
| Côte d'Ivoire | 2006 | 40 | MICS |  | Paraguay | 2012 | 11 | ONS |
| Côte d'Ivoire | 2012 | 30 | DHS |  | Paraguay | 2016 | 6 | MICS |
| Dominican Rep. | 1991 | 21 | NNS |  | Peru | 1992 | 37 | DHS |
| Dominican Rep. | 1996 | 14 | ONS |  | Peru | 1996 | 32 | DHS |
| Dominican Rep. | 2000 | 8 | NNS |  | Peru | 2000 | 31 | DHS |
| Dominican Rep. | 2002 | 12 | MICS |  | Peru | 2005 | 29 | DHS |
| Dominican Rep. | 2006 | 11 | MICS |  | Peru | 2008 | 28 | DHS |
| Dominican Rep. | 2007 | 10 | DHS |  | Peru | 2009 | 24 | DHS |
| Dominican Rep. | 2013 | 7 | DHS |  | Peru | 2010 | 23 | DHS |
| Ecuador | 1986 | 40 | MICS |  | Peru | 2011 | 20 | DHS |
| Ecuador | 1998 | 28 | DHS |  | Peru | 2012 | 18 | DHS |
| Ecuador | 2004 | 29 | ONS |  | Peru | 2013 | 18 | DHS |
| Ecuador | 2006 | 26 | DHS |  | Peru | 2014 | 15 | DHS |
| Ecuador | 2013 | 25 | DHS |  | Peru | 2015 | 15 | DHS |
| Ecuador | 2014 | 24 | DHS |  | Peru | 2016 | 13 | DHS |
| Egypt | 1991 | 35 | DHS |  | Philippines | 1987 | 45 | NNS |
| Egypt | 1993 | 31 | NNS |  | Philippines | 1990 | 43 | NNS |
| Egypt | 1996 | 35 | ONS |  | Philippines | 1992 | 41 | NNS |
| Egypt | 2000 | 24 | ONS |  | Philippines | 1993 | 39 | NNS |
| Egypt | 2003 | 20 | ONS |  | Philippines | 1998 | 38 | NNS |
| Egypt | 2005 | 24 | NNS |  | Philippines | 2003 | 34 | NNS |
| Egypt | 2008 | 31 | ONS |  | Philippines | 2008 | 32 | NNS |
| Egypt | 2014 | 22 | NNS |  | Philippines | 2011 | 34 | NNS |
| El Salvador | 1988 | 37 | DHS |  | Philippines | 2014 | 30 | NNS |
| El Salvador | 1993 | 32 | DHS |  | Philippines | 2015 | 33 | NNS |
| El Salvador | 1998 | 32 | DHS |  | Rwanda | 1992 | 57 | DHS |
| El Salvador | 2003 | 25 | DHS |  | Rwanda | 2000 | 48 | DHS |
| El Salvador | 2008 | 21 | DHS |  | Rwanda | 2005 | 51 | DHS |
| El Salvador | 2014 | 14 | DHS |  | Rwanda | 2011 | 44 | DHS |
| Ethiopia | 2000 | 58 | DHS |  | Rwanda | 2015 | 38 | DHS |
| Ethiopia | 2005 | 50 | NNS |  | Senegal | 1993 | 30 | DHS |
| Ethiopia | 2011 | 44 | NNS |  | Senegal | 1996 | 29 | MICS |
| Ethiopia | 2014 | 40 | ONS |  | Senegal | 2000 | 26 | MICS |
| Ethiopia | 2016 | 38 | ONS |  | Senegal | 2005 | 20 | DHS |
| Gabon | 2001 | 26 | ONS |  | Senegal | 2011 | 27 | DHS |
| Gabon | 2012 | 17 | MICS |  | Senegal | 2012 | 16 | NNS |
| Gambia | 2000 | 24 | DHS |  | Senegal | 2013 | 19 | DHS |
| Gambia | 2006 | 28 | DHS |  | Senegal | 2014 | 19 | DHS |
| Gambia | 2010 | 23 | DHS |  | Senegal | 2015 | 21 | DHS |
| Gambia | 2012 | 21 | ONS |  | Senegal | 2016 | 17 | DHS |
| Gambia | 2013 | 25 | DHS |  | Sierra Leone | 1989 | 41 | NNS |
| Georgia | 1999 | 16 | DHS |  | Sierra Leone | 1990 | 41 | NNS |
| Georgia | 2000 | 11 | DHS |  | Sierra Leone | 2000 | 36 | MICS |
| Georgia | 2005 | 15 | MICS |  | Sierra Leone | 2005 | 45 | MICS |
| Georgia | 2009 | 11 | MICS |  | Sierra Leone | 2008 | 37 | DHS |
| Ghana | 1988 | 37 | MICS |  | Sierra Leone | 2010 | 44 | MICS |
| Ghana | 1999 | 31 | NNS |  | Sierra Leone | 2013 | 38 | DHS |
| Ghana | 2003 | 35 | DHS |  |  |  |  |  |
| Ghana | 2006 | 28 | MICS |  |  |  |  |  |
| Ghana | 2008 | 28 | DHS |  |  |  |  |  |
| Ghana | 2011 | 23 | MICS |  |  |  |  |  |
| Ghana | 2014 | 19 | DHS |  |  |  |  |  |
|  |  |  |  |  |  |  |  |  |

## Table A1. Stunting prevalence and data sources (Cont’d)

|  |  |  |  |  |  |  |  |  |
| --- | --- | --- | --- | --- | --- | --- | --- | --- |
| Country | Year | Stunting prevalence (%) | Stunting data source |  | Country | Year | Stunting prevalence (%) | Stunting data source |
| Guatemala | 1995 | 55 | DHS |  | Sri Lanka | 2007 | 17 | DHS |
| Guatemala | 1999 | 53 | DHS |  | Sri Lanka | 2009 | 19 | NNS |
| Guatemala | 2000 | 51 | NNS |  | Sri Lanka | 2016 | 17 | DHS |
| Guatemala | 2002 | 55 | ONS |  | Sudan | 2010 | 34 | MICS |
| Guatemala | 2009 | 52 | ONS |  | Sudan | 2014 | 38 | MICS |
| Guatemala | 2015 | 47 | DHS |  | Suriname | 2000 | 14 | MICS |
| Guinea | 1999 | 31 | DHS |  | Suriname | 2006 | 11 | MICS |
| Guinea | 2005 | 39 | DHS |  | Suriname | 2010 | 9 | MICS |
| Guinea | 2008 | 40 | NNS |  | Tajikistan | 2005 | 33 | MICS |
| Guinea | 2012 | 34 | NNS |  | Tajikistan | 2007 | 39 | LSMS |
| Guinea | 2016 | 32 | MICS |  | Tajikistan | 2012 | 27 | DHS |
| Guinea-Bissau | 2000 | 34 | MICS |  | Tanzania | 1992 | 50 | DHS |
| Guinea-Bissau | 2006 | 48 | MICS |  | Tanzania | 1996 | 50 | DHS |
| Guinea-Bissau | 2010 | 32 | MICS |  | Tanzania | 1999 | 48 | DHS |
| Guinea-Bissau | 2014 | 28 | MICS |  | Tanzania | 2005 | 44 | DHS |
| Guyana | 1981 | 26 | NNS |  | Tanzania | 2009 | 43 | LSMS |
| Guyana | 1997 | 14 | NNS |  | Tanzania | 2010 | 42 | DHS |
| Guyana | 2000 | 14 | MICS |  | Tanzania | 2011 | 35 | LSMS |
| Guyana | 2007 | 18 | MICS |  | Tanzania | 2013 | 37 | LSMS |
| Guyana | 2009 | 19 | DHS |  | Tanzania | 2014 | 35 | NNS |
| Guyana | 2014 | 11 | MICS |  | Tanzania | 2016 | 34 | DHS |
| Haiti | 1995 | 37 | DHS |  | Thailand | 1993 | 21 | NNS |
| Haiti | 2000 | 29 | DHS |  | Thailand | 1995 | 18 | NNS |
| Haiti | 2006 | 30 | DHS |  | Thailand | 2006 | 16 | MICS |
| Haiti | 2012 | 22 | DHS |  | Thailand | 2013 | 16 | MICS |
| Honduras | 1987 | 43 | NNS |  | Thailand | 2016 | 11 | MICS |
| Honduras | 1992 | 43 | ONS |  | Timor-Leste | 2008 | 57 | ONS |
| Honduras | 1994 | 46 | ONS |  | Timor-Leste | 2010 | 58 | DHS |
| Honduras | 2001 | 35 | ONS |  | Timor-Leste | 2010 | 58 | DHS |
| Honduras | 2006 | 30 | DHS |  | Timor-Leste | 2013 | 51 | NNS |
| Honduras | 2012 | 23 | DHS |  | Togo | 2006 | 29 | MICS |
| India | 1979 | 75 | NNS |  | Togo | 2008 | 27 | NNS |
| India | 1990 | 66 | NNS |  | Togo | 2010 | 30 | MICS |
| India | 1992 | 65 | NNS |  | Togo | 2014 | 28 | DHS |
| India | 1993 | 57 | ONS |  | Tunisia | 1975 | 42 | NNS |
| India | 1997 | 49 | NNS |  | Tunisia | 1995 | 31 | PAPFAM |
| India | 2006 | 48 | DHS |  | Tunisia | 1997 | 12 | NNS |
| India | 2014 | 41 | ONS |  | Tunisia | 2000 | 17 | MICS |
| India | 2016 | 38 | DHS |  | Tunisia | 2006 | 9 | MICS |
| Indonesia | 1995 | 48 | MICS |  | Tunisia | 2012 | 10 | MICS |
| Indonesia | 2000 | 42 | SVLLS |  |  |  |  |  |
| Indonesia | 2001 | 42 | SVLLS |  |  |  |  |  |
| Indonesia | 2004 | 29 | ONS |  |  |  |  |  |
| Indonesia | 2007 | 40 | NNS |  |  |  |  |  |
| Indonesia | 2010 | 39 | ONS |  |  |  |  |  |
| Indonesia | 2013 | 36 | ONS |  |  |  |  |  |

## Table A1. Stunting prevalence and data sources (Cont’d)

|  |  |  |  |  |  |  |  |  |
| --- | --- | --- | --- | --- | --- | --- | --- | --- |
| Country | Year | Stunting prevalence (%) | Stunting data source |  | Country | Year | Stunting prevalence (%) | Stunting data source |
| Iran, Islamic Rep. | 1995 | 24 | MICS |  | Turkey | 1993 | 24 | DHS |
| Iran, Islamic Rep. | 1998 | 20 | NNS |  | Turkey | 1998 | 19 | DHS |
| Iran, Islamic Rep. | 2011 | 7 | ONS |  | Turkey | 2004 | 15 | ONS |
| Iraq | 1991 | 28 | NNS |  | Turkey | 2008 | 13 | ONS |
| Iraq | 2000 | 28 | MICS |  | Turkey | 2014 | 10 | ONS |
| Iraq | 2003 | 34 | NNS |  | Turkmenistan | 2000 | 28 | DHS |
| Iraq | 2004 | 20 | ONS |  | Turkmenistan | 2006 | 19 | MICS |
| Iraq | 2006 | 27 | MICS |  | Turkmenistan | 2016 | 11 | MICS |
| Iraq | 2011 | 22 | MICS |  | Uganda | 1989 | 48 | DHS |
| Jamaica | 1978 | 17 | NNS |  | Uganda | 1995 | 45 | DHS |
| Jamaica | 1989 | 12 | LSMS |  | Uganda | 2001 | 45 | DHS |
| Jamaica | 1991 | 12 | LSMS |  | Uganda | 2006 | 38 | DHS |
| Jamaica | 1992 | 14 | LSMS |  | Uganda | 2011 | 33 | DHS |
| Jamaica | 1993 | 13 | LSMS |  | Uganda | 2012 | 34 | LSMS |
| Jamaica | 1994 | 12 | LSMS |  | Uganda | 2016 | 29 | DHS |
| Jamaica | 1995 | 10 | LSMS |  | Uzbekistan | 2002 | 25 | DHS |
| Jamaica | 1996 | 11 | LSMS |  | Uzbekistan | 2006 | 20 | MICS |
| Jamaica | 1997 | 9 | LSMS |  | Venezuela | 1982 | 9 | NNS |
| Jamaica | 1998 | 10 | LSMS |  | Venezuela | 1987 | 7 | NNS |
| Jamaica | 1999 | 6 | LSMS |  | Venezuela | 1990 | 19 | SVLLS |
| Jamaica | 2000 | 7 | LSMS |  | Venezuela | 1991 | 18 | SVLLS |
| Jamaica | 2001 | 7 | LSMS |  | Venezuela | 1992 | 18 | SVLLS |
| Jamaica | 2002 | 8 | LSMS |  | Venezuela | 1993 | 17 | SVLLS |
| Jamaica | 2004 | 5 | LSMS |  | Venezuela | 1994 | 18 | SVLLS |
| Jamaica | 2006 | 8 | LSMS |  | Venezuela | 1995 | 19 | SVLLS |
| Jamaica | 2007 | 5 | LSMS |  | Venezuela | 1996 | 19 | SVLLS |
| Jamaica | 2009 | 7 | LSMS |  | Venezuela | 1997 | 20 | SVLLS |
| Jamaica | 2010 | 6 | LSMS |  | Venezuela | 1998 | 19 | SVLLS |
| Jamaica | 2012 | 7 | LSMS |  | Venezuela | 1999 | 18 | SVLLS |
| Jamaica | 2014 | 6 | ONS |  | Venezuela | 2000 | 17 | SVLLS |
| Jordan | 1990 | 20 | DHS |  | Venezuela | 2001 | 17 | NNS |
| Jordan | 1997 | 11 | DHS |  | Venezuela | 2002 | 18 | NNS |
| Jordan | 2002 | 12 | DHS |  | Venezuela | 2003 | 18 | NNS |
| Jordan | 2009 | 8 | DHS |  | Venezuela | 2004 | 17 | NNS |
| Jordan | 2012 | 8 | DHS |  |  |  |  |  |
|  |  |  |  |  |  |  |  |  |

## Table A1. Stunting prevalence and data sources (Cont’d)

|  |  |  |  |  |  |  |  |  |
| --- | --- | --- | --- | --- | --- | --- | --- | --- |
| Country | Year | Stunting prevalence (%) | Stunting data source |  | Country | Year | Stunting prevalence (%) | Stunting data source |
| Kazakhstan | 1999 | 13 | DHS |  | Venezuela | 2005 | 16 | NNS |
| Kazakhstan | 2006 | 17 | MICS |  | Venezuela | 2006 | 16 | NNS |
| Kazakhstan | 2011 | 13 | MICS |  | Venezuela | 2007 | 16 | SVLLS |
| Kazakhstan | 2015 | 8 | MICS |  | Venezuela | 2008 | 15 | NNS |
| Kenya | 1993 | 40 | DHS |  | Venezuela | 2009 | 13 | NNS |
| Kenya | 1994 | 41 | NNS |  | Viet Nam | 1984 | 64 | NNS |
| Kenya | 2000 | 41 | MICS |  | Viet Nam | 1989 | 61 | NNS |
| Kenya | 2003 | 36 | DHS |  | Viet Nam | 1993 | 61 | LSMS |
| Kenya | 2006 | 40 | ONS |  | Viet Nam | 1994 | 53 | NNS |
| Kenya | 2009 | 35 | DHS |  | Viet Nam | 1998 | 42 | NNS |
| Kenya | 2014 | 26 | DHS |  | Viet Nam | 1999 | 44 | SVLLS |
| Lao PDR | 1993 | 54 | ONS |  | Yemen | 2011 | 47 | NNS |
| Lao PDR | 1994 | 53 | NNS |  | Yemen | 2013 | 46 | DHS |
| Lao PDR | 2000 | 48 | ONS |  | Zambia | 1992 | 46 | DHS |
| Lao PDR | 2006 | 48 | MICS |  | Zambia | 1997 | 49 | DHS |
| Lao PDR | 2012 | 44 | DHS/MICS |  | Zambia | 2002 | 53 | DHS |
| Lebanon | 1996 | 17 | PAPFAM |  | Zambia | 2007 | 46 | DHS |
| Lebanon | 2004 | 17 | PAPFAM |  | Zambia | 2014 | 40 | DHS |
| Lesotho | 1992 | 39 | NNS |  | Zimbabwe | 1988 | 31 | DHS |
| Lesotho | 2000 | 53 | MICS |  | Zimbabwe | 1999 | 34 | DHS |
| Lesotho | 2005 | 45 | DHS |  | Zimbabwe | 2006 | 35 | DHS |
| Lesotho | 2010 | 39 | DHS |  | Zimbabwe | 2009 | 35 | MICS |
| Lesotho | 2014 | 33 | DHS |  | Zimbabwe | 2011 | 32 | DHS |
| Liberia | 1976 | 43 | NNS |  | Zimbabwe | 2015 | 27 | DHS |
| Liberia | 2000 | 45 | NNS |  |  |  |  |  |
| Liberia | 2007 | 40 | DHS |  |  |  |  |  |
| Liberia | 2010 | 39 | ONS |  |  |  |  |  |
| Liberia | 2013 | 32 | DHS |  |  |  |  |  |
|  |  |  |  |  |  |  |  |  |

## Table A2. Sample composition by region and decades covered

|  |  |  |  |  |  |  |
| --- | --- | --- | --- | --- | --- | --- |
|  |  | Stunting data coverage by decade (%) | | | | |
| Region | Share of total sample (%) | 1970-1979 | 1980-1989 | 1990-1999 | 2000-2010 | 2011-2017 |
| Central Asia | 2 | 0 | 0 | 8 | 58 | 33 |
| Eastern Asia | 3 | 0 | 6 | 33 | 39 | 22 |
| LAC | 26 | 1 | 9 | 30 | 38 | 21 |
| Northern Africa | 6 | 3 | 3 | 37 | 37 | 20 |
| South-eastern Asia | 12 | 0 | 7 | 23 | 41 | 30 |
| Southern Asia | 7 | 6 | 3 | 26 | 31 | 34 |
| Sub-Saharan Africa | 37 | 0 | 5 | 18 | 39 | 38 |
| Western Asia | 7 | 0 | 0 | 30 | 46 | 24 |
| Note: LAC = Latin America and the Caribbean consisting of Central America, South America and Caribbean countries. | | | | | |  |

## Table A3. Correlation coefficients between key variables

| Variable | (1) | (2) | (3) | (4) | (5) | (6) | (7) | (8) | (9) | (10) | (11) | (12) | (13) | (14) |
| --- | --- | --- | --- | --- | --- | --- | --- | --- | --- | --- | --- | --- | --- | --- |
| 1. Stunting prevalence | 1 |  |  |  |  |  |  |  |  |  |  |  |  |  |
| 1. Milk supply | -0.50*** | 1 |  |  |  |  |  |  |  |  |  |  |  |  |
| 1. Non-dairy ASF supply | -0.38*** | 0.15*** | 1 |  |  |  |  |  |  |  |  |  |  |  |
| 1. Fruit supply | -0.18*** | 0.12** | 0.50*** | 1 |  |  |  |  |  |  |  |  |  |  |
| 1. Vegetable supply | -0.31*** | 0.12** | 0.44*** | 0.49*** | 1 |  |  |  |  |  |  |  |  |  |
| 1. Legume & nuts supply | 0.27*** | 0.02 | -0.12** | 0.18*** | -0.05 | 1 |  |  |  |  |  |  |  |  |
| 1. Starchy roots supply | 0.01 | -0.37*** | -0.10* | 0.19*** | 0.01 | -0.05 | 1 |  |  |  |  |  |  |  |
| 1. Cereals supply | 0.12** | 0.02 | 0.57*** | 0.45*** | 0.33*** | 0.29*** | -0.12** | 1 |  |  |  |  |  |  |
| 1. GDP per capita | -0.73*** | 0.55*** | 0.45*** | 0.33*** | 0.30*** | -0.24*** | -0.11* | -0.06 | 1 |  |  |  |  |  |
| 10. Child dependency ratio | 0.58*** | -0.29*** | -0.42*** | -0.19*** | -0.49*** | 0.38*** | 0.10* | 0.06 | -0.56*** | 1 |  |  |  |  |
| 11. Access to electricity | -0.54*** | 0.35*** | 0.39*** | 0.28*** | 0.49*** | -0.35*** | -0.26*** | -0.03 | 0.68*** | -0.69*** | 1 |  |  |  |
| 12. Female labor | 0.28*** | -0.35*** | -0.02 | -0.08 | -0.19*** | -0.03 | 0.37*** | -0.03 | -0.37*** | 0.10* | -0.42*** | 1 |  |  |
| 13. Improved sanitation | -0.49*** | 0.32*** | 0.25*** | 0.08 | 0.26*** | -0.27*** | -0.24*** | -0.11** | 0.50*** | -0.47*** | 0.58*** | -0.30*** | 1 |  |
| 14. Improved water | -0.53*** | 0.37*** | 0.27*** | 0.20*** | 0.33*** | -0.28*** | -0.30*** | -0.10* | 0.61*** | -0.57*** | 0.74*** | -0.40*** | 0.60*** | 1 |

## Figure A1. Cross-sectional associations between stunting prevalence, per capita milk supply and per capita GDP using a trimmed sample


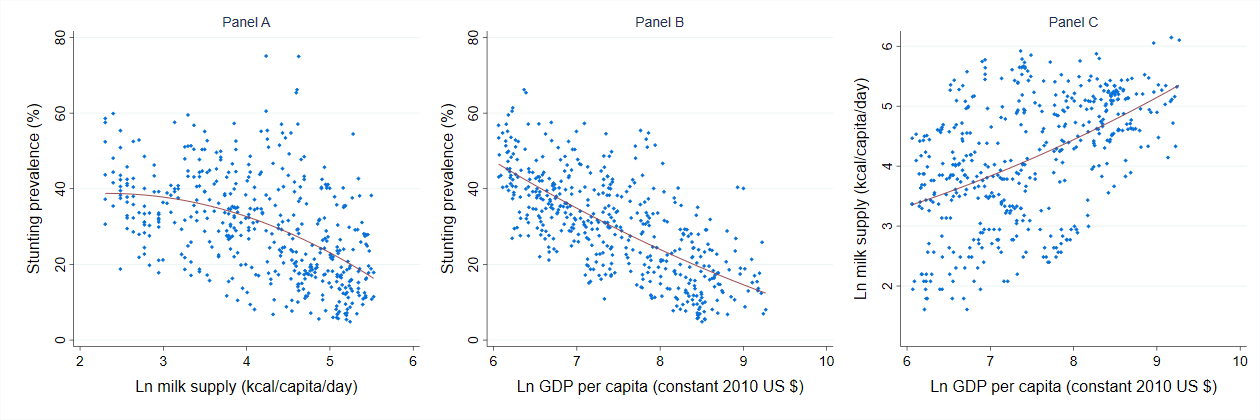


Note: Bivariate associations are based on a subset of countries for whom the value of the respective right-hand side variable is between the 5th and 95th percentile (trimmed sample).

## Table A4. Country ranking based on compound average growth rate (CAGR) of milk supply

|  |  |  |  |  |
| --- | --- | --- | --- | --- |
| Country | Region | First and last year of stunting | CAGR of milk supply (%) | AARR of stunting prevalence (%) |
| Timor-Leste | South-eastern Asia | 08_13 | 17.08 | -2.30 |
| Viet Nam | South-eastern Asia | 84_15 | 9.67 | -3.04 |
| China | Eastern Asia | 87_13 | 6.67 | -5.80 |
| Myanmar | South-eastern Asia | 00_16 | 6.06 | -2.04 |
| Maldives | Southern Asia | 95_09 | 5.91 | -4.02 |
| Ethiopia | SSA | 00_16 | 5.65 | -2.50 |
| Gambia | SSA | 00_13 | 5.20 | 0.15 |
| Armenia | Western Asia | 98_16 | 5.06 | -2.60 |
| Azerbaijan | Western Asia | 00_13 | 4.54 | -2.33 |
| Guinea | SSA | 99_16 | 4.43 | 0.25 |
| Thailand | South-eastern Asia | 93_16 | 4.36 | -2.99 |
| Benin | SSA | 01_14 | 3.68 | -0.46 |
| Cape Verde | SSA | 83_94 | 3.68 | -0.77 |
| Togo | SSA | 06_14 | 3.66 | -0.83 |
| Indonesia | South-eastern Asia | 95_13 | 3.63 | -1.54 |
| Sri Lanka | Southern Asia | 07_16 | 3.10 | 0.00 |
| Ghana | SSA | 88_14 | 3.08 | -2.54 |
| Lao PDR | South-eastern Asia | 93_12 | 2.99 | -1.01 |
| Nicaragua | LAC | 93_12 | 2.94 | -2.79 |
| Mali | SSA | 01_15 | 2.94 | -2.36 |
| Chad | SSA | 97_15 | 2.93 | -0.63 |
| El Salvador | LAC | 88_14 | 2.82 | -3.73 |
| Mauritius | SSA | 85_95 | 2.81 | -6.73 |
| Guinea-Bissau | SSA | 00_14 | 2.68 | -1.44 |
| Yemen | Western Asia | 92_13 | 2.61 | -0.39 |
| Morocco | Northern Africa | 87_11 | 2.48 | -3.01 |
| Egypt | Northern Africa | 91_14 | 2.39 | -1.94 |
| Pakistan | Southern Asia | 91_13 | 2.29 | -0.87 |
| India | Southern Asia | 79_16 | 2.27 | -1.83 |
| Bangladesh | Southern Asia | 97_14 | 2.26 | -2.89 |
| Uganda | SSA | 89_16 | 2.08 | -1.83 |
| Colombia | LAC | 80_10 | 1.96 | -2.65 |
| Kazakhstan | Central Asia | 99_15 | 1.94 | -3.05 |
| Haiti | LAC | 95_12 | 1.79 | -3.02 |
| Namibia | SSA | 92_13 | 1.78 | -2.12 |
| Tanzania, United Republic of | SSA | 92_16 | 1.74 | -1.53 |
| Rwanda | SSA | 92_15 | 1.67 | -1.71 |
| Brazil | LAC | 96_14 | 1.67 | 0.18 |
| Algeria | Northern Africa | 92_13 | 1.59 | -3.16 |
| Guyana | LAC | 81_14 | 1.59 | -2.55 |
| Peru | LAC | 92_16 | 1.48 | -4.28 |
| Tunisia | Northern Africa | 75_12 | 1.47 | -3.76 |
| Zambia | SSA | 92_14 | 1.38 | -0.66 |
| Lesotho | SSA | 92_14 | 1.35 | -0.73 |
| Honduras | LAC | 87_12 | 1.30 | -2.56 |
| Nigeria | SSA | 90_17 | 1.19 | -0.41 |
| Paraguay | LAC | 90_16 | 1.14 | -4.46 |
| Ecuador | LAC | 86_14 | 1.14 | -1.83 |

## Table A4. Country ranking based on compound average growth rate (CAGR) of milk supply (Cont’d)

|  |  |  |  |  |
| --- | --- | --- | --- | --- |
| Country | Region | First and last year of stunting | CAGR of milk supply (%) | AARR of stunting prevalence (%) |
| Panama | LAC | 80_08 | 1.09 | -0.87 |
| Turkey | Western Asia | 93_14 | 1.05 | -4.03 |
| Burkina Faso | SSA | 93_16 | 0.97 | -1.59 |
| Niger | SSA | 85_16 | 0.93 | -0.25 |
| Mexico | LAC | 88_15 | 0.81 | -2.83 |
| Turkmenistan | Central Asia | 00_16 | 0.75 | -5.45 |
| Mongolia | Eastern Asia | 97_13 | 0.74 | -6.39 |
| Bolivia | LAC | 81_16 | 0.72 | -3.11 |
| Jamaica | LAC | 78_14 | 0.59 | -2.79 |
| Dominican Republic | LAC | 91_13 | 0.49 | -4.82 |
| Jordan | Western Asia | 90_12 | 0.48 | -4.19 |
| Uzbekistan | Central Asia | 02_06 | 0.43 | -5.82 |
| Guatemala | LAC | 95_15 | 0.35 | -0.85 |
| Nepal | Southern Asia | 75_17 | 0.23 | -1.73 |
| Congo (Brazzaville) | SSA | 87_15 | 0.19 | -1.63 |
| Liberia | SSA | 00_11 | 0.00 | -2.62 |
| Lebanon | Western Asia | 00_13 | 0.00 | -0.50 |
| Sierra Leone | SSA | 89_13 | 0.00 | -0.38 |
| Central African Republic | SSA | 96_04 | 0.00 | -0.77 |
| Georgia | Western Asia | 99_09 | -0.09 | -3.48 |
| Tajikistan | Central Asia | 05_12 | -0.15 | -2.95 |
| Kenya | SSA | 93_14 | -0.16 | -2.01 |
| Sudan | Northern Africa | 93_14 | -0.17 | -0.04 |
| Iraq | Western Asia | 91_11 | -0.62 | -1.11 |
| Cameroon | SSA | 91_14 | -0.64 | 0.02 |
| Iran, Islamic Republic of | Southern Asia | 95_11 | -0.71 | -7.67 |
| Angola | SSA | 01_16 | -0.81 | -2.05 |
| Zimbabwe | SSA | 88_15 | -1.02 | -0.50 |
| Venezuela (Bolivarian Republic) | LAC | 82_09 | -1.02 | 1.32 |
| Mauritania | SSA | 88_15 | -1.32 | -1.34 |
| Suriname | LAC | 00_10 | -1.38 | -4.62 |
| Gabon | SSA | 01_12 | -1.72 | -3.72 |
| Botswana | SSA | 96_08 | -1.74 | -1.62 |
| Malawi | SSA | 92_16 | -1.87 | -1.62 |
| Philippines | South-eastern Asia | 87_15 | -1.95 | -1.04 |
| Malaysia | South-eastern Asia | 99_16 | -2.04 | 0.00 |
| Mozambique | SSA | 95_11 | -2.20 | -2.06 |
| Cambodia | South-eastern Asia | 96_14 | -2.23 | -3.23 |
| Côte d'Ivoire | SSA | 86_12 | -2.83 | 1.10 |
| Sao Tome and Principe | SSA | 06_14 | -2.97 | -6.28 |
| Madagascar | SSA | 92_04 | -3.03 | -1.16 |
| Senegal | SSA | 93_16 | -3.05 | -2.38 |
| Belize | LAC | 06_16 | -3.69 | -3.76 |
| Note: LAC = Latin America and the Caribbean. SSA = Sub-Saharan Africa. Survey years show the last two digits of the first and last years of available nutrition data. CAGR means compound annual growth rate. AARR means average annual rate of reduction. | | | | |

## Table A5. Fixed effects estimates of the association between milk supply and stunting prevalence (unweighted regressions)

|  |  |  |  |  |
| --- | --- | --- | --- | --- |
|  | (1) | (2) | (3) | (4) |
| Ln milk supply (k/c/d) | -6.37*** | -6.25*** | -3.13*** | -3.13** |
|  | (1.22) | (1.14) | (1.17) | (1.21) |
| Ln non-dairy animal-source foods (k/c/d) |  | -0.27 | -0.14 | -0.07 |
|  |  | (0.99) | (0.82) | (0.84) |
| Ln fruit supply (k/c/d) |  | -0.48 | -0.72 | -0.90 |
|  |  | (0.69) | (0.60) | (0.60) |
| Ln vegetable supply (k/c/d) |  | -2.22 | -1.19 | -1.28 |
|  |  | (1.52) | (1.60) | (1.58) |
| Ln legume and nuts supply (k/c/d) |  | 0.78 | 0.72 | 0.81 |
|  |  | (0.53) | (0.55) | (0.55) |
| Ln starchy roots supply (k/c/d) |  | -1.21 | -0.63 | -0.49 |
|  |  | (1.15) | (1.02) | (1.02) |
| Ln cereals supply (k/c/d) |  | 0.82** | 0.54* | 0.53* |
|  |  | (0.33) | (0.29) | (0.28) |
| Ln GDP per capita (constant 2010 US $) |  |  | -6.22*** | -5.75*** |
|  |  |  | (1.65) | (1.58) |
| Population growth rate |  |  | 0.25 | 0.17 |
|  |  |  | (0.24) | (0.23) |
| Ln child dependency ratio |  |  | 11.18*** | 10.75*** |
|  |  |  | (3.17) | (2.93) |
| Ln access to electricity (% of population) (rural) |  |  | -1.22 | -0.95 |
|  |  |  | (0.83) | (0.85) |
| Ln of female labor force participation rate (15-24) (%) |  |  |  | 1.58 |
|  |  |  |  | (2.31) |
| Ln of population using improved sanitation (%) |  |  |  | -0.30 |
|  |  |  |  | (0.33) |
| Ln of population using improved drinking water (%) |  |  |  | -4.40 |
|  |  |  |  | (2.86) |
| Number of observations | 524 | 524 | 524 | 524 |
| R2 between | 0.13 | 0.20 | 0.48 | 0.47 |
| R2 within | 0.63 | 0.65 | 0.69 | 0.70 |
| R2 overall | 0.627 | 0.650 | 0.693 | 0.697 |
| Number of observations | 524 | 524 | 524 | 524 |
| R2 | 0.910 | 0.916 | 0.928 | 0.931 |
| Note: Reported are unweighted fixed effects regressions. Ln means natural logarithm. k/c/d stands for kilocalories per capita per day. Robust standard errors clustered at the country level in parentheses. All columns control for subregion-specific time trends. *** p<0.01, ** p<0.05, * p<0.1. | | | | |

## Table A6. Fixed effects estimates of the association between milk supply, wasting prevalence, and under-5 mortality

|  |  |  |
| --- | --- | --- |
|  | Wasting | Mortality |
|  | (1) | (2) |
| Ln milk supply (k/c/d) | -0.26 | -0.89 |
|  | (0.57) | (4.64) |
| Ln non-dairy animal-source foods (k/c/d) | 0.67* | 1.58 |
|  | (0.35) | (3.59) |
| Ln fruit supply (k/c/d) | -0.74* | -8.78* |
|  | (0.41) | (4.57) |
| Ln vegetable supply (k/c/d) | 0.51 | -1.26 |
|  | (0.65) | (5.62) |
| Ln legume and nuts supply (k/c/d) | -0.09 | 1.34 |
|  | (0.24) | (2.41) |
| Ln starchy roots supply (k/c/d) | -0.34 | -4.44 |
|  | (0.71) | (4.88) |
| Ln cereals supply (k/c/d) | 0.04 | 2.58* |
|  | (0.13) | (1.55) |
| Ln GDP per capita (constant 2010 US $) | -1.08 | -26.91*** |
|  | (0.73) | (9.39) |
| Ln population growth rate | -0.18 | -4.69*** |
|  | (0.12) | (1.00) |
| Ln child dependency ratio | 3.70** | -34.51* |
|  | (1.42) | (19.61) |
| Ln access to electricity | 0.30 | -4.40 |
|  | (0.29) | (4.34) |
| Ln of female labor force participation rate (15-24) (%) | 0.05 | -15.08 |
|  | (0.88) | (9.81) |
| Ln of population using improved sanitation (%) | -0.10 | -1.33 |
|  | (0.15) | (2.01) |
| Ln of population using improved drinking water (%) | 0.20 | -32.12** |
|  | (1.25) | (14.91) |
| Number of observations | 524 | 524 |
| R2-squared between | 0.16 | 0.23 |
| R2-squared within | 0.21 | 0.77 |
| R2-overall | 0.214 | 0.770 |
| Note: Reported are fixed effects regressions where the dependent variable is under-5 wasting (column 1) and mortality (column 2). Ln means natural logarithm. k/c/d stands for kilocalories per capita per day. Robust standard errors clustered at the country level in parentheses. All columns control for subregion-specific time trends. *** p<0.01, ** p<0.05, * p<0.1 | | |
